# A predictive ensemble classifier for the gene expression diagnosis of ASD at ages 1 to 4 years

**DOI:** 10.1038/s41380-022-01826-x

**Published:** 2022-10-20

**Authors:** Bokan Bao, Javad Zahiri, Vahid H. Gazestani, Linda Lopez, Yaqiong Xiao, Raphael Kim, Teresa H. Wen, Austin W. T. Chiang, Srinivasa Nalabolu, Karen Pierce, Kimberly Robasky, Tianyun Wang, Kendra Hoekzema, Evan E. Eichler, Nathan E. Lewis, Eric Courchesne

**Affiliations:** 1 Autism Center of Excellence, Department of Neuroscience, University of California San Diego, La Jolla, CA USA; 2 Department of Pediatrics, University of California San Diego, La Jolla, CA USA; 3 Bioinformatics and Systems Biology Program, University of California San Diego, La Jolla, CA USA; 4 Department of Bioengineering, University of California San Diego, La Jolla, CA USA; 5 Center for Language and Brain, Shenzhen Institute of Neuroscience, Shenzhen, China; 6 Department of Computer Science, University of North Carolina, Chapel Hill, NC USA; 7 Renaissance Computing Institute, University of North Carolina at Chapel Hill, Chapel Hill, NC USA; 8 Department of Genetics, University of North Carolina at Chapel Hill, Chapel Hill, NC 27514 US; 9 School of Information and Library Science, University of North Carolina at Chapel Hill, Chapel Hill, NC 27599 USA; 10 Carolina Health and Informatics Program, University of North Carolina at Chapel Hill, Chapel Hill, NC 27599 USA; 11 Department of Medical Genetics, Center for Medical Genetics, Peking University Health Science Center, 100191 Beijing, China; 12 Neuroscience Research Institute, Peking University; Key Laboratory for Neuroscience, Ministry of Education of China & National Health Commission of China, 100191 Beijing, China; 13 Department of Genome Sciences, University of Washington School of Medicine, Seattle, WA 98195 USA; 14 Howard Hughes Medical Institute, University of Washington, Seattle, WA 98195 USA

**Keywords:** Neuroscience, Diagnostic markers, Autism spectrum disorders, Molecular biology

## Abstract

Autism Spectrum Disorder (ASD) diagnosis remains behavior-based and the median age of diagnosis is ~52 months, nearly 5 years after its first-trimester origin. Accurate and clinically-translatable early-age diagnostics do not exist due to ASD genetic and clinical heterogeneity. Here we collected clinical, diagnostic, and leukocyte RNA data from 240 ASD and typically developing (TD) toddlers (175 toddlers for training and 65 for test). To identify gene expression ASD diagnostic classifiers, we developed 42,840 models composed of 3570 gene expression feature selection sets and 12 classification methods. We found that 742 models had AUC-ROC ≥ 0.8 on both Training and Test sets. Weighted Bayesian model averaging of these 742 models yielded an ensemble classifier model with accurate performance in Training and Test gene expression datasets with ASD diagnostic classification AUC-ROC scores of 85–89% and AUC-PR scores of 84–92%. ASD toddlers with ensemble scores above and below the overall ASD ensemble mean of 0.723 (on a scale of 0 to 1) had similar diagnostic and psychometric scores, but those below this ASD ensemble mean had more prenatal risk events than TD toddlers. Ensemble model feature genes were involved in cell cycle, inflammation/immune response, transcriptional gene regulation, cytokine response, and PI3K-AKT, RAS and Wnt signaling pathways. We additionally collected targeted DNA sequencing smMIPs data on a subset of ASD risk genes from 217 of the 240 ASD and TD toddlers. This DNA sequencing found about the same percentage of SFARI Level 1 and 2 ASD risk gene mutations in TD (12 of 105) as in ASD (13 of 112) toddlers, and classification based only on the presence of mutation in these risk genes performed at a chance level of 49%. By contrast, the leukocyte ensemble gene expression classifier correctly diagnostically classified 88% of TD and ASD toddlers with ASD risk gene mutations. Our ensemble ASD gene expression classifier is diagnostically predictive and replicable across different toddler ages, races, and ethnicities; out-performs a risk gene mutation classifier; and has potential for clinical translation.

## Introduction

ASD is a prenatal [1–16], highly heritable disorder [17] that considerably impacts a child’s ability to perceive and react to social information [18–20]. Despite this prenatal and strongly genetic beginning, robust and replicable early-age biological ASD diagnostic markers useful at the individual level have not been found. Indeed, ASD diagnosis remains behavior-based and the median age of the first diagnosis remains at ~52 months [21–24], which is nearly 5 years after its first trimester origin. The long delay between ASD’s prenatal onset and eventual diagnosis is a missed opportunity for treatment. Moreover, the heterogeneity of ASD genetics and clinical characteristics impose barriers to identifying early-age molecular diagnostics that accurately diagnose the majority of those with this heterogeneous disorder [25]. Thus, there is a need for early-age molecular diagnostics of ASD that robustly surmount this heterogeneity obstacle.

Since ASD’s heritability is 81% [17], initial attempts have focused on genetics to develop clinically useful biomarkers for precision medicine and causal explanations for ASD pathogenesis. While syndromic risk mutations have been described for >200 genes in ASD [16, 26, 27], each occurs only rarely in ASD. For 80–90% of patients, such mutations are not found. Thus, an estimated 80% or more of ASD individuals are considered ‘idiopathic’, wherein little is known about the genes and/or environmental factors causing their disorder. In this idiopathic majority of ASD, the risk is likely associated with many inherited common and rare risk variants in each individual child. Studies of polygenic ASD risk found that the combined effect of genetic risk variants in case-control studies accounts for less than 7.5% of the risk variance [28]; ASD polygenic risk scores substantially overlap with controls [29–32]; and, because of this substantial overlap, polygenic risk scores are not clinically diagnostic or prognostic for individuals, nor are they explanatory for the majority of ASD. Thus, DNA-based mutations or polygenic risk scores may not yet be useful for the many idiopathic ASD subjects at the clinical diagnostic level.

RNA biomarkers have been sought using blood gene expression in >35 ASD studies since 2006 [33–43], but many studies have been underpowered, older-aged, clinically heterogeneous, and/or lacking validation test datasets. Some early genetics researchers rejected blood-based biomarkers believing that ASD-relevant dysregulated gene expression must be restricted to the brain. Recent ASD genetics have reversed this view: The earliest prenatal drivers of deviant ASD development are, in fact, broadly expressed regulatory genes, a large percentage of which are active in non-brain organs and tissues such as blood leukocytes as well as in the prenatal brain [1–3, 33, 34, 37, 38, 43, 44]. Broadly expressed genes that constitute most ASD risk genes are upregulated in early prenatal life and impact multiple stages of prenatal brain development from 1^st^ and 2^nd^ trimester proliferation and neurogenesis to neurite outgrowth and synaptogenesis in the 3^rd^ trimester. These genes disrupt gene expression in signaling pathways such as PI3K-AKT, RAS-ERK, Wnt and insulin receptor pathways, which further disrupt prenatal functions [1–3, 33, 34, 37, 38, 43, 44].

In ASD 1 to 4 year-olds, leukocyte gene expression in these pathways is significantly dysregulated [45]. The degree of pathway dysregulation was correlated with ASD social symptom severity and were validated in ASD neural progenitors and neurons [45]. Broadly expressed genes in leukocytes from ASD toddlers are also associated with hypoactive brain responses to language and atypical cortical patterning, dysregulation of ASD and language relevant genes, and poor language outcomes [46, 47]. Thus, leukocyte gene expression holds the potential for the objective identification of molecular subtypes of ASD. In analyses of leukocyte gene co-expression, ASD-associated module eigengene values were significantly correlated with abnormal early brain growth and enriched in genes related to cell cycle, translation, and immune networks and pathways. These gene sets are very accurate classifiers of ASD vs. typically developing toddlers (TD) [34]. Studies and reviews of the ASD blood gene expression literature [33–43, 45] show dysregulated gene expression in a number of pathways and processes, including PI3K-AKT-mTOR, RAS signaling pathways, ribosomal translation signal, cell cycle, neurogenesis, gastrointestinal disease, immune/inflammation, interferon signaling, and the KEGG natural killer cytotoxicity pathway.

Leukocyte gene expression offers a non-invasive and clinically practicable avenue for understanding aspects of ASD cell biology, including those that could be ASD-relevant, ASD-specific, robust, and ASD-diagnostic or -prognostic. However, for clinical translational potential of leukocyte transcriptomics to lead to robust and rigorous classifiers, high standards for verifying such classifiers should be implemented.

**Fig. 1:**
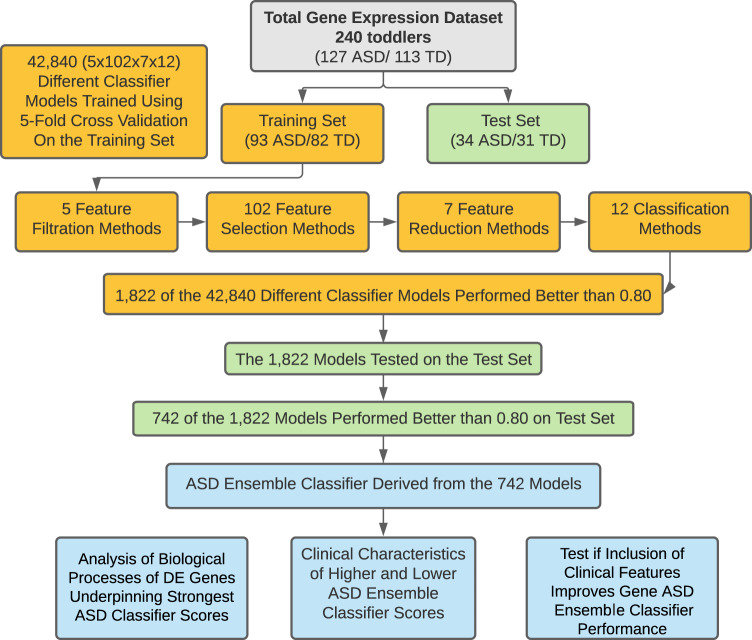
Overview of the analysis platform. The total gene expression dataset was split into a Training set with 175 subjects and a Test set with 65 subjects. Our platform tested 42,840 different models, with each model a combination of 1 feature filtration method, 1 feature selection method, 1 feature reduction method and 1 classification method (total different combinations = 5 × 102 × 7 × 12 = 42,840 models). Models processed the input datasets and returned classification scores. In total, 742 models had classification scores ≥0.8 AUC-ROC in both Training and Test sets were used to build the final ensemble classifier model.

Thus, we developed, operationalized, and tested a rigorous analytic pipeline to identify molecular diagnostic classifiers for ASD using leukocyte gene expression. Using additional clinical data, we verified that our composite gene expression classifier was unbiased against common confounding factors (age, race and ethnicity). Using this platform on leukocyte transcriptomics from male ASD and typically developing (TD) toddlers at ages 1–4 years old, we systematically analyzed the classification performance of 42,840 different models composed of 3,570 different feature selection sets and 12 commonly-used classification methods (Fig. 1 and Supplementary Fig. 1). Through this, we developed a predictive ensemble diagnostic classifier of male ASD toddlers.

Additionally, using targeted DNA sequencing of the coding regions for sets of ASD and neurodevelopmental disorder risk genes using single-molecule molecular inversion probes (smMIPs) [48, 49], we examined the diagnostic classifier value of presence or absence of a subset of ASD risk gene mutations in our ASD and TD subjects and whether toddlers with ASD risk gene mutations differ in classifier expression from those without such mutations.

## Methods

### Participant recruitment and clinical evaluation

Participants in this study included 240 male toddlers ages 1–4 years (Table 1, Supplementary Table 1). About 70% of toddlers were recruited from the general population using an early screening, detection, and diagnosis strategy called the Get SET Early procedure [50]. Using this approach, toddlers who failed a broadband screen, i.e., the CSBS IT Checklist [51], at 12, 18 or 24 month well-baby visits in the general pediatric community settings, were referred to our center for a comprehensive diagnostic and psychometric evaluation. The remaining subjects were obtained by general community referrals and evaluated in the identical way. Median ages were ASD 2.3 years and TD 1.4 years. All toddlers received a battery of standardized psychometric tests by experienced Ph.D.-level psychologists, including the Autism Diagnostic Observation Schedule (ADOS; Module T, 1 or 2) [52], the Mullen Scales of Early Learning [53], and the Vineland Adaptive Behavior Scales [54]. Testing sessions routinely lasted 4 h in one day or occurred across 2 separate days. Toddlers younger than 30 months upon initial clinical evaluation were followed longitudinally approximately every 9–12 months until final confirmation diagnosis at ages 2 to 4 years; Table 1 shows demographic and subject characteristics at final confirmation ages. 127 toddlers were diagnosed ASD, and 113 were TD. Research procedures were approved by the Institutional Review Board of the University of California, San Diego. Parents of subjects underwent Informed Consent Procedures with a psychologist or study coordinator at the time of their child’s enrollment.

**Table 1:** Subjects demographics.

	Training set			Test set		
	ASD	TD	ASD vs TD *p* value	ASD	TD	ASD vs TD *p* value
Number of Subjects	93	82	0.406	34	31	0.71
Age of last Visit in	50.8 ± 28.8	34.5 ± 8.4	<0.001	47.4 ± 28.0	39.2 ± 15.3	0.144
months						
Mullen Scales of Early Learning						
Visual Reception	40.1 ± 13.3	60.3 ± 10.9	<0.001	38.8 ± 15.4	55.1 ± 9.2	<0.001
Fine Motor	34.8 ± 11.4	54.4 ± 9.5	<0.001	36.9 ± 13.9	52.8 ± 8.9	<0.001
Receptive Language	32.2 ± 13.1	53.3 ± 8.1	<0.001	29.0 ± 16.6	52.4 ± 7.6	<0.001
Expressive Language	30.7 ± 15.8	54.4 ± 9.6	<0.001	28.5 ± 16.8	49.8 ± 8.1	<0.001
Early Learning Composite	73.6 ± 18.5	111.1 ± 13.3	<0.001	71.9 ± 21.0	105.0 ± 11.1	<0.001
Vineland Adaptive Behavior Scales						
Communication	82.0 ± 17.5	104.9 ± 10.5	<0.001	79.5 ± 17.8	100.4 ± 9.6	<0.001
Daily Living	83.7 ± 12.8	103.0 ± 10.2	<0.001	84.0 ± 13.0	99.9 ± 10.6	<0.001
Socialization	80.5 ± 13.0	106.3 ± 10.9	<0.001	79.1 ± 9.8	99.4 ± 11.0	<0.001
Motor Skills	87.8 ± 10.9	103.1 ± 10.4	<0.001	88.5 ± 10.5	98.8 ± 8.5	<0.001
Adaptive Behavior	80.8 ± 13.0	105.0 ± 9.7	<0.001	79.9 ± 11.6	99.2 ± 10.3	<0.001
Autism Diagnostic Observation Schedule						
ADOS SA/CoSo Score	14.3 ± 3.4	2.2 ± 2.0	<0.001	13.3 ± 4.3	2.8 ± 1.9	<0.001
ADOS RRB Score	3.8 ± 1.5	0.3 ± 0.6	<0.001	3.1 ± 1.5	0.5 ± 0.6	<0.001
ADOS Total Score	18.1 ± 4.1	2.4 ± 2.1	<0.001	16.4 ± 4.7	3.3 ± 2.2	<0.001

*ADOS* autism diagnostic observation schedule, *ASD* autism spectrum disorder, *CoSo* communication social score, *M/F* male/female, *RRB* restricted and repetitive behavior, *SA* social affect.

### Targeted sequencing data from ASD and TD subjects

For 112 of the 127 ASD and 105 of the 113 TD study subjects, we also had targeted sequencing data by smMIPs from prior studies aimed at detecting rare severe mutations in autism and neurodevelopmental disorder risk genes; that study was from our Center’s collaboration with the Eichler Lab [48, 49]. Two sets of neurodevelopmental disorders and ASD risk genes were used for targeted sequencing (See Supplementary Table 2). The ASD significant variants in our ASD toddlers had been previously reported, but here we additionally report ASD significant variants in our TD toddlers. More than 87% of the ASD toddlers (83 out of 93 and 29 out of 34 ASDs in the Training and Test datasets, respectively), and 92% of the TD toddlers (74 out of 82 and all 31 TDs in the Training and Test datasets, respectively) were tested for mutations. Rare (MAF < 0.01%) severe missense mutation with a combined annotation-dependent depletion (CADD) score ≥30 (MIS30) and likely gene-disruptive (LGD, including splicing donor or acceptor, frameshift, and stop-gained) mutations were considered for further analysis. Among the 105 TD toddlers, 12 had SFARI Level 1 or 2 ASD risk gene mutations and among the 112 ASD toddlers, 13 had such mutations. One of these ASD had two ASD risk gene mutations. Thus, among the 217 subjects, a total of 25 subjects carried ASD risk gene mutations (26 genes). The two-sided independent T-test was performed to test the ensemble score distribution difference between subjects with or without mutations.

### Blood sample collection for gene expression analyses

Blood samples were collected from each subject during clinical evaluation visits. To monitor health status, the temperature of each toddler was taken using an ear digital thermometer immediately preceding the blood draw. When the temperature was higher than 99 Fahrenheit, the blood draw was re-scheduled for a later visit. Moreover, the blood draw was not taken if a toddler had some illness (e.g., cold or flu), as observed by us or stated by parents. We collected four to six milliliters of blood into ethylenediaminetetraacetic-coated tubes from all toddlers. Leukocytes in the blood samples were captured and stabilized by LeukoLOCK filters (Ambion) and were immediately placed in a − 20 °C freezer. Total RNA was extracted following standard procedures and manufacturer’s instructions (Ambion).

### Summary of main steps in design and analyses of the RNA data from the 240 study subjects

Figure 1 outlines the main design and analysis steps, and Supplementary Fig. 1 provided details of the feature engineering. The 240 subjects were divided into a Training dataset of 175 subjects and a Test set of 65 subjects. The training dataset was used to build gene expression classifiers and the Test set was held out and later used to test the classifiers. High-performing classifiers evaluated by the Test set were used to build a single, final ensemble classifier, which was a Bayesian averaging model of all top-performing classifiers. The performance of this ensemble classifier was then measured on Training and Test subjects; DE genes underlying its accurate performance were identified and pathway and process enrichment determined; and clinical characteristics across classifier scores were examined. Lastly, post hoc exploratory analyses were performed to test whether including specific social behavioral and prenatal features might improve overall performance.

### Microarray data processing

Gene expression of subject RNA samples was assayed using the Illumina HT-12 platform. Arrays were scanned with the Illumina BeadArray Reader and read into Illumina GenomeStudio software (version 1.1.1). Raw Illumina probe intensities were converted to expression values using the lumi package [55]. We employed a three-step procedure to filter for probes with reliable expression levels. First, we only retained probes that met the detection *p* < 0.05 cut-off threshold in at least 3 samples. Second, we required probes to have expression levels above the 95^th^ percentile of negative probes in at least 50% of samples. The probes with detection *p* > 0.1 across all samples were selected as negative probes and their expression levels were pooled together to estimate the 95th percentile expression level. Third, for genes represented by multiple probes, we considered the probe with the highest mean expression level across our dataset, after quantile normalization of the data. These criteria led to the selection of 14,312 coding genes as expressed in our leukocyte transcriptome data, which highly overlaps with the reported estimate of 14,555 protein-coding genes (chosen based on unique Entrez gene IDs) for whole blood by the GTEx consortium [56].

### Building the classifier platform on the training dataset

The pipeline ran five-fold cross-validations. At the beginning of each iteration, the pipeline held out 20% of samples and used the remaining 80% of samples for hyper-parameter selection, feature selection, and classifier training. In the first step (Supplementary Fig. 1), feature filtration, five methods were used, including no (no action), cov (remove 50% of features with the smaller coefficient of variation), var (remove 50% of features with smaller variance), cov_var (remove 50% of features with the smaller coefficient of variation and then remove 50% of features with smaller variance in the rest), varImportance (keep only the 25% of features with the highest variance).

The second step, feature selection, included 102 methods, which were composed of seven groups; although conceptually similar, each using different approaches. These seven groups are no (no action), grn [57] (genetic regulatory network), z-score, select [58], svm [59], GSEA [60], DE-analysis [61] (see Supplementary Method 1).

The third step was feature reduction. Seven methods were used: no (no feature reduction), WGCNA [62], logisticFwd, SIS [63], principal component regression (PCR) [64], partial least squares regression (PLSR) [65], canonical powered partial least squares (CPPLS) [65] (see Supplementary Method 1). After three steps, up to 1320 gene routes were created that can be used in the classification step.

The classification step exploited 12 classifiers, including reg (linear model), logReg [66] (logistic regression), lda [66] (Linear Discriminant Analysis), qda [66] (Quadratic Discriminant Analysis), ridgeReg [67] (GLM with ridge regularization), lassoReg [67] (GLM with lasso regularization), ridgeLogReg [67] (logistic regression with ridge regularization), lassoLogReg [67] (logistic regression with lasso regularization), elasticNetLogReg [67] (logistic regression with elastic net regularization), boosting [68] (Generalized Boosted Regression Modeling with Bernoulli distribution), randomForest [69] (random forest) and bagging [69] (random forests with bagging to reduce the complexity). After training a classifier, the diagnostic ability was evaluated by AUC-PR (precision-recall) curve and AUC-ROC (Receiver operating characteristic) curve [70, 71].

For every possible combination of the 5 feature filtration, 102 feature selection, 7 feature reduction, and 12 classification routes, we made a total of 42,840 different classifier models.

### Label permutation data

To generate the randomized background, we shuffled the diagnostic label of the Training dataset and randomly separated the data into training/validation segments (85%/15%). Then we performed the fivefold cross-validation on the permuted dataset.

### Bayesian model averaging to create a single transcriptomic ensemble classifier

The training models that had 0.80 or higher AUC-ROC scores were tested on the Test dataset. Then, the models that had an AUC-ROC ≥ 0.80 were used with Bayesian Model Averaging (BMA) to create a single ensemble classifier. The ensemble score was the sum of weighted predictions of selected models. The weight was the mathematical average of the square of (AUC-ROC value minus 0.7). In a model selection, we used training data D to select a good model M (according to a score) to predict a targeted outcome T of interest based on patient features X, namely, P(T|X, M). BMA was based on the notion of averaging over a set of possible models and weighting the prediction of each model according to its probability given training data D, as shown in equations.

- $${{{{{{{\mathrm{p}}}}}}}}\left( {{{{{{{{\mathrm{T|X}}}}}}}}} \right) = \mathop {\sum}\nolimits_{{{{{{{{\mathrm{m}}}}}}}}_{{{{{{{\mathrm{i}}}}}}}}} {{{{{{{{\mathrm{p}}}}}}}}\left( {{{{{{{{\mathrm{M}}}}}}}}_{{{{{{{\mathrm{i}}}}}}}}{{{{{{{\mathrm{|X}}}}}}}}} \right){{{{{{{\mathrm{p}}}}}}}}\left( {{{{{{{{\mathrm{T|X}}}}}}}},{{{{{{{\mathrm{M}}}}}}}}_{{{{{{{\mathrm{i}}}}}}}}} \right)}$$

M is the model, T is the prediction and X is the data.

- $${{{{{{{\mathrm{p}}}}}}}}\left( {{{{{{{{\mathrm{M}}}}}}}}_{{{{{{{\mathrm{i}}}}}}}}{{{{{{{\mathrm{|X}}}}}}}}} \right) = \frac{{{{{{{{{\mathrm{AUC}}}}}}}}\_{{{{{{{\mathrm{ROC}}}}}}}}_{{{{{{{\mathrm{i}}}}}}}} - 0.7}}{{\mathop {\sum}\nolimits_{{{{{{{\mathrm{j}}}}}}}} {\left( {{{{{{{{\mathrm{AUC}}}}}}}}\_{{{{{{{\mathrm{ROC}}}}}}}}_{{{{{{{\mathrm{i}}}}}}}} - 0.7} \right)} }}$$

The ensemble scores of the independent dataset were calculated based on the same model built. The scores are then rescaled to 0 and 1.

- $$ensembleScore_i = \frac{{ensembleScore_i - min\left( {ensembleScore} \right)}}{{max\left( {ensembleScore} \right) - min\left( {ensembleScore} \right)}}$$

### Biological processes enriched by differentially expressed genes

We additionally conducted differential expression (DE) analysis on ASD subjects with ensemble scores below-the-mean vs. all TD subjects. The Limma package [61, 72] was then applied on quantile-normalized data for differential expression analysis in which moderated t-statistics were calculated by robust empirical Bayes methods. We used adjusted *p* < 0.01 (Benjamin–Hochberg) and log Fold Change >0.1 to select genes and generate the volcano plot. The Gene Ontology (GO) enrichment was conducted using g:Profiler [73] (https://biit.cs.ut.ee/gprofiler/gost) with 12,695 protein-coding genes (12695/14132 gene features) as background (g:Profiler, advanced option/statistical domain scope: Custom; custom over annotated genes). We only checked the “GO biological process” and KEGG terms of size 15–1500 in the biological process. The threshold was “Significance threshold: B-H FDR < 0.1”. Then the terms were clustered with REVIGO [74], ordered with *p* (http://revigo.irb.hr/). The connections across terms were visualized by the Cytoscape 3.8.2 [75].

### Post-hoc analysis on common confounding factors

The post-hoc analysis further verified that the classifier scores were stable across different age groups. The optbin R package was used to determine optimal age breakpoints for ASD and TD groups; age bins were 0 to 20, 20 to 31, and 31 to 49 months. Games-Howell test [76] was performed to compare the classifier score between TD or ASD groups in each of the three age bins (FDR adjusted *p* value < 0.05).

The one-way ANOVA test [77] was conducted to test if statistically significant differences existed across three ethnicities and seven race groups for ASD subjects. For ethnicity, toddlers from ASD and TD were labeled as “Hispanic or Latino”, “Not Hispanic or Latino” and “Unknown”. For races, toddlers from ASD and TD were labeled as “Caucasian”, “Caucasian/Asian”, “African American”, “Asian”, “Pacific Islander”, “Other”, “Unknown”.

## Results

### ASD risk gene mutation-based diagnostic classification of ASD vs TD

Targeted sequencing by smMIPs was performed on 217 (112 ASD and 105 TD) out of the 240 (127 ASD and 113 TD) toddlers in this study (see “Methods” and Supplementary Table 3). Analyses found 12 TD toddlers with missense or LGD mutations in SFARI (https://gene.sfari.org/) Level 1 or 2 ASD risk genes including: *ANK3*, *CACNA2D3*, *CLCN4*, *CTTNBP2*, *CUL7*, *DIP2A*, *DLG4*, *HECTD4*, *LRP2*, *LZTR1*, *MYH9*, and *NAV2*. Analyses found 13 ASD toddlers with missense or LGD mutations in SFARI (https://gene.sfari.org/) Level 1 or 2 ASD risk genes: *CACNA2D3*, *CHD2*, *DIP2A*, *DSCAM*, *KATNAL2*, *LRP2*, *MYH9*, *NCKAP1*, *NTNG1*, *PHF2*, *RELN*, *STXBP5*, *UNC80*, and *ZC3H4* (one subject had two mutations). To assess the power of using this mutation information alone in discriminating ASD from TD, we did a classification according to the presence/absence of the missense or LGD mutations in SFARI Level 1 or 2 ASD risk genes. More precisely, ASD toddlers with and without mutations were considered as true positive and false negative, respectively; and TD toddlers with and without mutations were considered as false positive and true negative, respectively. This mutation-based classification performed at a chance level, 49% (50% being chance), with precision (positive predictive value) of 52%, and recall (sensitivity) of 10%. In this mutation-based classification, a small number of TDs were falsely called ASD and a large number of ASD toddlers were falsely called TD.

### Development of a robust transcriptomic classifier platform with diverse feature engineering and classification methods

Next, we used blood transcriptomic data from the 240 ASD and TD study toddlers to develop a diagnostic classifier. To identify potential transcriptome biomarkers in a Training sample of 175 of the 240 ASD and TD toddlers (Table 1), we developed a platform that examined the classification power of the blood transcriptomic data by systematically exploring the performance of 42,840 possible models composed of 3570 different feature selection routes, followed by 12 classification methods (see “Methods”). The platform started with removing genes with low variation across samples. Next, features that differentiate between ASD and TD subjects at expression or co-expression levels were selected using a suite of 102 feature selection methods. Third, to avoid overfitting, we reduced the number of features by collapsing expression data from the correlating genes. Finally, we trained 12 different classifiers for each selected feature set. To evaluate the performance of each of the 3570 feature selection routes and the 12 classification methods, we iterated the process 5 times while holding out 20% of samples and using the remaining 80% of samples for hyper-parameter selection, feature selection, and classifier training. Thus, each of the 42,840 models started with a “route” that consisted of 1 filtration method, 1 selection method, 1 reduction method, and ended with 1 classification method, and all possible combinations of the 5 filtration, 102 selection, 7 reduction and 12 classification methods were used. The platform reports the average performance of each of the 42,840 models across the 5 held-out folds as measured by area under the receiver operating characteristic curve (AUC-ROC) and area under the precision-recall curve (AUC-PR).

### Diverse pipelines successfully classify ASD vs TD

Since the feature selection methods depended on the characteristics of training transcriptome datasets, some routes were not able to find qualified features in all five iterations of the validation. Therefore, the platform successfully classified the data in 15,840 out of 42,840 different ways, including 1320 different routes out of 3570 for feature selection and 12 different classification methods (Supplementary Table 4 and Supplementary Fig. 2). From 15,840 trained models, 1822 (11.5%) models showed classification AUC-ROC > 0.8 with the max AUC-ROC of 0.856. Moreover, 1508 of the 1822 models also exhibited an AUC-PR > 0.8.

**Fig. 2:**
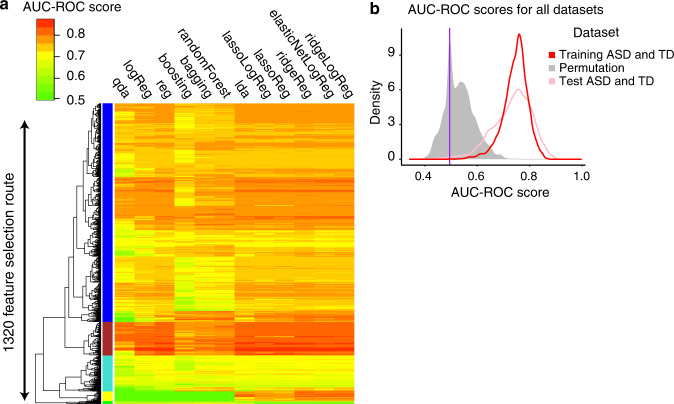
A classification platform was developed to robustly identify the biomarkers for the early diagnosis of ASD. **a** AUC-ROC classifier scores were computed for each of the 42,840 model results from the Training dataset. The AUC-ROC values were based on the average performance of each model across 5 iterations, with 20% of samples being held out each time. **b** In total, 1822 models with AUC-ROC scores ≥0.80 were then tested on the held out Test dataset. Permuting the sample labels (i.e., ASD and TD) further supported the validity of the signal.

These 1822 models performed well due to their feature routes and were robust to variations in the data or the model. For example, we observed a subset of 175 feature routes (colored with a brown band in Fig. 2a) that performed consistently well across different classifiers with a mean AUC-ROC of 0.81. Additionally, these 1822 high-performing models worked similarly well across all five held-out datasets with a mean range of 0.13 and variance of 0.02 (Supplementary Fig. 3). Furthermore, different models that largely overlapped in their feature selection routes also worked well across different classifier methods (Supplementary Fig. 5).

To further verify that the performance of these 1822 models was not due to chance alone, we generated five separate randomized datasets by shuffling the sample labels (i.e., ASD or TD) from the Training dataset. We next ran the platform on each of the five datasets independently (see “Methods”). Importantly, the platform identified zero models out of 1822 with AUC-ROC and AUC-PR > 0.8 across the five datasets, respectively, suggesting that the accurate performance of the 1822 models was not due to chance.

**Fig. 3:**
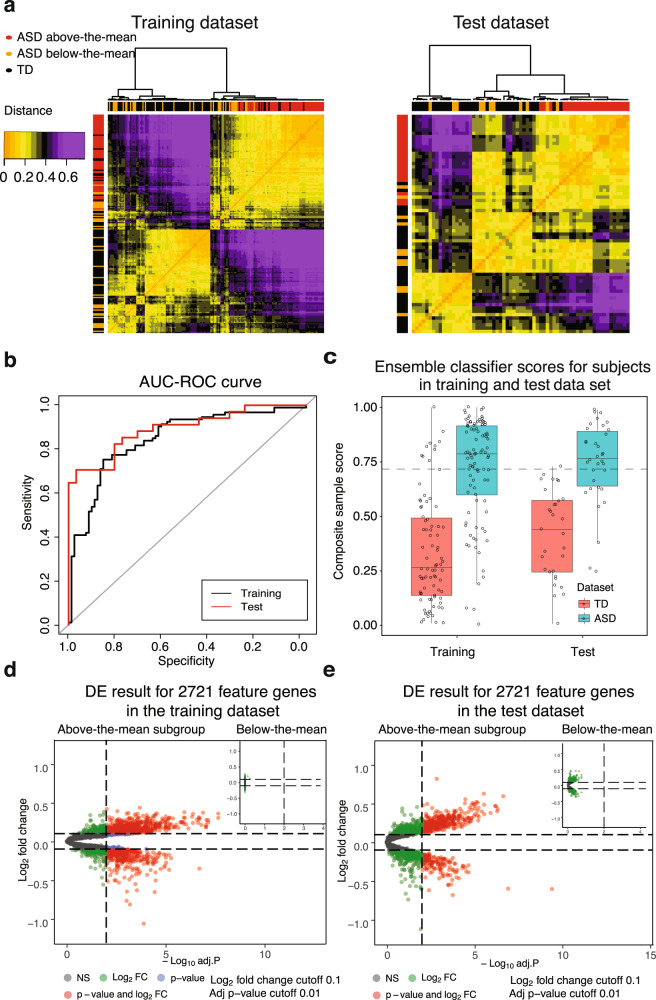
Blood transcriptome ASD subtypes were identified by our classification platform. **a** The clustering table of subjects in Training and Test dataset based on the 742 models classification score similarity (distance = “Euclidean” and method = “ward.2d”). ASD and TD subjects showed distinct classification patterns. The red, orange and black bars on the sides represented above-the-mean ASD, below-the-mean ASD and TD subjects, respectively (the mean is the dashed line in Fig. 3c). The orange and purple colors represented the gradient of dissimilarity between subjects based on their classification scores. (b) The AUC-ROC results on the ensemble classification model generated by the Bayesian model averaging approach. **c** Ensemble classifier model scores for ASD and TD individuals in Training and Test datasets. The ASD group mean was 0.723 and the TD group mean was 0.359. **d**, **e** The differential expression analysis of 2721 protein-coding feature genes. The volcano plots showed the adjusted *p* value (cutoff = 0.01) vs. log fold changes (cutoff = 0.1) of genes in the above-the-mean to TD subjects and below-the-mean subjects to TD subjects in the Training dataset and Test dataset.

We evaluated the performance of the 1822 high-performing models on the Test dataset of *N* = 65 ASD and TD toddlers. Of the 1822 models with AUC-ROC > 0.8 in the Training dataset, 742 models (40%; Fisher’s Exact Test *p* < 2.2 × 10^−16^) also had an AUC-ROC > 0.8 for the Test dataset. These 742 heterogeneous predictive models involved 125 different feature routes and 2721 gene features (Fig. 3a, see Supplementary Result 1, 2).

### Randomized data can be erroneously “classified” at reasonable AUC-ROC levels

There were 1822 models that reached a high AUC-ROC value in the Training dataset. However, the question remained whether this range was significantly different from the AUC-ROC values that one could obtain from trying to classify subjects after randomizing their final diagnosis. To test this, we permuted the sample labels (i.e., ASD and TD) for all subjects in our Training set and ran the pipeline to test all feature engineering and classification methods. Importantly, we tested all 42,840 candidate models and found the median AUC-ROC score was 0.5101 with the 95th CI (0.42–0.65) on the randomized samples. As expected, only rare chance instances of good “classification” occurred. The fact that chance alone could lead to a rare “good classification” score for a single model, was a cautionary signal that literature reports of unvalidated and unreplicable single high-performance classifiers could be due to chance (see “Methods”, Fig. 2b).

### Bayesian model averaging of the 742 predictive models to create a single transcriptomic ensemble classifier

To build a composite ensemble model that combined the 742 models that had AUC-ROC values of 0.80 on both Training and held-out Test sets, we used Bayesian model averaging (BMA). The ensemble model produced a single composite classification score by calculating weighted predictions from 742 models (see “Methods”). Scores ranged from 0 to 1 with 0 being the highest certainty in TD status and 1 being the highest certainty in ASD status. With this ensemble model, the AUC-ROC score was 84.67% and 89.18% for Training and Test datasets, respectively (Fig. 3b) and AUC-PR was 84.33% and 92.11% for the Training and Test datasets, respectively. These values were significantly higher than the naive Random Forest baseline model (see Supplementary Result 3) with 72.32% AUC-ROC (ROC.test *p* < 10^−44^).

We calculated the median of ensemble classification scores for all ASD toddlers in the Training and Test datasets. The overall ASD group median classifier score was 0.781 and the overall TD group median score was 0.303 (Fig. 3c). To test for group differences in scores and possible age effects, we used multiple linear regression. The independent variables were diagnosed group, age and their interaction. The dependent variable was the ensemble classification score. Based on the coefficients in the model, we found a significant effect of group (coefficient, *p* = 0.0011) but non-significant effects of age (coefficient, *p* = 0.056) and group by age interaction (coefficient, group:age, *p* = 0.76).

### Classifier scores not significantly affected by age, ethnicity, race differences

**Fig. 4:**
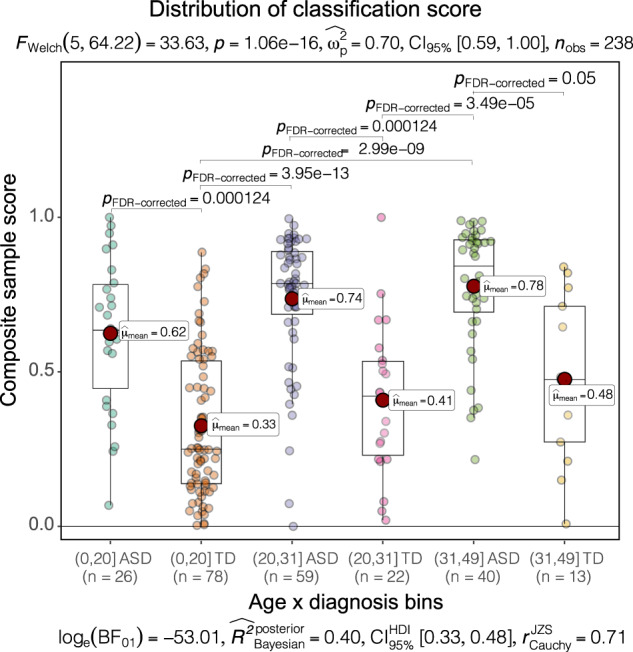
The distribution of classification scores. 240 subjects were partitioned into 6 groups. Games-Howell tests were performed to compare the group difference and only significant comparisons were shown. The classification scores were significantly different only in the ASD vs TD diagnostic group comparison.

To further examine possible bias toward the age effects on group classification, we stratified subjects into three age bins of 0 to 20, 20 to 31, and 31 to 49 months and compared the classifier prediction performance on different bins (see “Method”, Fig. 4). Game-Howell test [76] showed there was no significant difference between classification scores for TD or ASD groups in each of the three age bins, and classification scores were significantly different only in the ASD vs. TD diagnostic group comparisons (FDR adjusted *p* value < 0.05) (Fig. 4). This further verified that potential confounding effects of age were excluded in the analysis.

Post-hoc examination of classifier scores in ASD groups showed there was no significant difference across three ethnicity groups (“Hispanic or Latino”, “not Hispanic or Latino”, “unknown”; one-way ANOVA, F = 0.899, *p* = 0.409) (Table 2). However, the differences appeared in TD groups (F = 3.7, *p* = 0.021). The same analysis was also conducted on races. Toddlers were labeled as “Caucasian”, “Caucasian/Asian”, “African American”, “Asian”, “Pacific Islander”, “Other”, “Unknown”. No significant difference of means in ASD was found across all race groups (One-way ANOVA test, F = 1.151, *p* = 0.337) (Table 3). The differences appeared in the TD group (F = 5.25, *p* = 9.03e–05) and seemed likely due to the small number of individuals in different race categories (Table 3). Both ethnicity and race analysis indicated that ASD molecular pathology is being consistently detected by our classifier.

**Table 2:** Statistics of ASD and TD’s classifier score for Hispanic/non-Hispanics.

Ethnicity	ASD		TD	
	Size	Mean (Std)	Size	Mean (Std)
Not Hispanic Latino	64	0.700 (0.253)	82	0.396 (0.248)
Hispanic and Latino	29	0.723 (0.197)	18	0.273 (0.220)
Unknown	34	0.765 (0.187)	13	0.245 (0.138)

**Table 3:** Statistics of ASD and TD’s classifier score for races.

Race	ASD		TD	
	Size	Mean (Std)	Size	Mean (Std)
Caucasian	65	0.675 (0.253)	73	0.364 (0.240)
Unknown	40	0.755 (0.180)	19	0.284 (0.175)
Caucasian/Asian	4	0.861 (0.150)	5	0.358 (0.212)
African American	5	0.799 (0.221)	4	0.508 (0.288)
Asian	8	0.789 (0.212)	9	0.421 (0.314)
Pacific Islander	3	0.795 (0.090)	2	0.185 (0.088)
Other	2	0.797 (0.005)	1	0.592 (null)

### Classifier scores not significantly affected by the presence or absence of ASD risk gene mutations

**Fig. 5:**
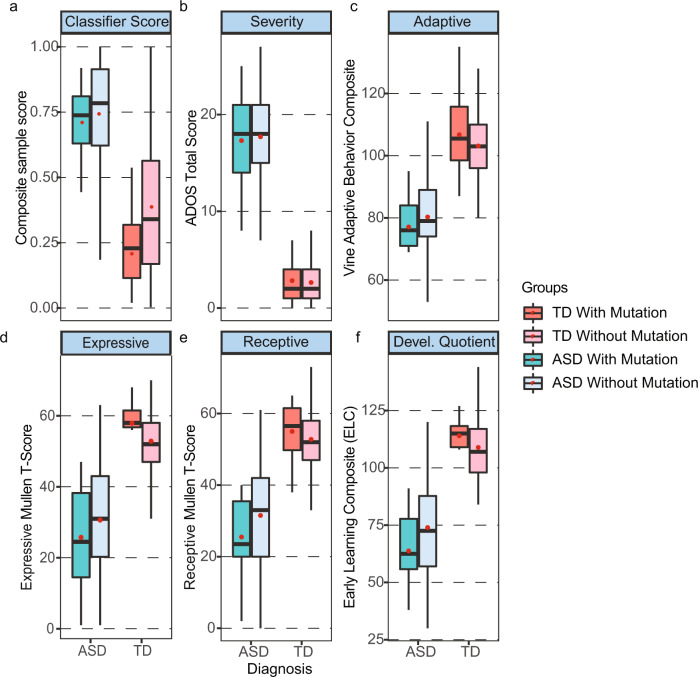
Comparison of ASD with and without ASD risk gene mutations and TD with and without ASD risk gene mutations. Shows (**a**) ensemble classifier gene expression scores, (**b**) ADOS scores (higher scores are more severe ASD symptoms), (**c**) Vineland adaptive behavior scores (average is 100 ± 15), and (**d**) the Mullen T-scores for Expressive and (**e**) Receptive language (average is 50 ± 10) as well as (**f**) the Mullen overall Developmental Quotient scores (average is 100 ± 15). There were no significant differences in any scores between toddlers with and without SFARI Level 1 or 2 ASD risk gene mutations for ASD toddlers and for TD toddlers. Thus, the presence of an ASD risk gene mutation conferred no clinical liability or difference in gene expression diagnostic score for ASD toddlers. TD toddlers with ASD gene mutations had slightly better cognitive scores than other typically developing toddlers without mutations, but differences were not significant. Red dots are the means and dark lines medians.

There was no significant difference in the ensemble classifier scores between ASD toddlers with and without mutations (median = 0.738 vs 0.784, mean = 0.715 vs. 0.724, Welch *t* test *p* = 0.875) (Fig. 5; Supplementary Table 3); 11 of the 13 ASD toddlers with risk mutations were correctly classified by the ensemble model. However, there was a difference in the ensemble classifier scores between TD toddlers with and without ASD risk gene mutations; in fact, TDs with mutations had lower composite scores than the other TDs (median = 0.229 vs 0.340, mean = 0.223 vs. 0.375, Welch *t* test *p* = 0.007) and robustly differed from the ASD composite score, median = 0.303 vs 0.781 (Fig. 5). Thus, the presence of ASD risk gene mutations conferred no liability on the composite score of TD toddlers, and 11 out of 12 TDs with mutations in risk genes were correctly classified as typical by our gene expression classifier. The ensemble classifier correctly differentially diagnosed 88% of the ASD and TD toddlers with ASD risk gene mutations.

In addition, TD subjects with and without SFARI Level 1 or 2 gene mutations did not differ significantly on any clinical test (ADOS, Vineland, Mullen), and, similarly, ASD subjects with and without gene mutations did not differ significantly on any clinical test (Fig. 5; Supplementary Fig. 7).

### Biological processes enriched by differentially expressed (DE) genes in ASD with higher vs. lower ASD ensemble classifier scores

DE gene analyses (see “Methods”) found 1186 DE genes for ASD toddlers with ensemble scores at or above the ASD group mean of 0.723, but no DE genes for those below the group mean (Fig. 3d, e). Of the 1,186 DE genes, 394 were in the top 500 feature genes selected by the 125 feature routes, and 700 of the 1,186 DE genes were in the first 1000 feature genes. This indicated that DE genes were strong drivers of successful ASD classification (Supplementary Table 5). Enrichment analyses of GO biological processes (see “Methods”) of these 1186 DE genes found Gene Ontology terms associated with mitotic cell cycle, inflammation/immune response, transcriptional gene regulation, and response to cytokine. Analyses of KEGG pathways using g:Profiler [73, 78] of these 1,186 DE genes found significant pathways included cell cycle (KEGG:hsa04110), PI3K-AKT (KEGG:hsa04151), RAS signaling pathways (KEGG:hsa04014), and Wnt signaling pathways (KEGG:hsa04310), which was consistent with our previous finding [45].

### Clinical characteristics associated with higher vs. lower ASD ensemble classifier scores

We compared clinical scores on the ADOS, Mullen, and Vineland for ASD toddlers with ensemble classifier scores at or above the ASD mean of 0.723 to the ASD toddlers with classifier scores below that mean. Diagnostic and psychometric scores were not significantly different between ASD subjects above and below this mean (Supplementary Fig. 8, Supplementary Table 6).

Next, we stratified ASD toddlers based on ADOS CoSo Total symptom severity and Mullen scores. Ensemble scores for ASD subjects above vs. below the group average ADOS severity and the group average Mullen means were not practically different (*p* = 0.59).

We also performed analogous stratifications within the TD Training group and found no ADOS or Mullen differences between higher or lower than the TD mean ensemble classifier score, nor differences in the ensemble scores of TD toddlers with high vs. lower diagnostic and psychometric scores (Supplementary Table 6).

### Prenatal characteristics associated with higher vs. lower ASD ensemble classifier scores

Among 127 ASD subjects, 124 had complete prenatal records. We selected the “hospitalization during trimester”, “surgery during trimester” and “confined to bed during trimester” as the risk factors; “nausea during trimester”, “morning sickness during trimester” and “swelling during trimester” as the control prenatal events. Fisher’s *t* tests were used to compare the prenatal risk factors across ASD toddlers with ensemble scores at or above the ASD group mean, below that mean, and TD toddlers. ASD toddlers with classifier scores at or above the ASD group mean of 0.723 had significantly fewer prenatal neurodevelopmental risk events, while ASD toddlers below the mean had disproportionately more prenatal risk scores than TD toddlers (Tables 4, 5). We tested if there was a different ratio of severe prenatal events that could potentially impact ASD development between these two ASD subgroups [79–81]. We found a similar rate of prenatal events between TD subjects and above-the-mean ASD subjects (Odds Ratio: 0.88, Fisher’s Exact Test *p* = 0.84). However, there was a significant enrichment of prenatal events among the below-the-mean ASD subjects compared to TD subjects (Odds Ratio: 2.78; Fisher’s Exact Test *p* = 0.013). As a negative control, prenatal events that are unlikely to affect ASD development were not enriched among ASD subjects with below the ASD mean ensemble score [81]. These results suggest the possible existence of different underlying etiological factors between ASD subjects with above vs. below the mean ASD ensemble classifier scores. (Tables 4, 5)

**Table 4:** Distribution of prenatal events among the three groups.

Severe prenatal events	ASD Above-the-mean	ASD Below-the-mean	TD
Total subjects (n)	79	45	107
Hospitalizations during pregnancy	5	5	6
Surgery during pregnancy	1	3	4
Confinement to bed during pregnancy	8	8	9
General Anesthesia during delivery	10	11	10
Total (%)	19 (24.1)	20 (44.4)	24 (22.4)
Negative control events			
Nausea	4	2	12
Morning sickness	42	18	64
Swelling	23	10	26
Total (%)	52 (65.8)	24 (53.3)	70 (65.4)

*ASD* at or above-the-mean ensemble score of 0.723, *ASD* below-the-mean ensemble score of 0.723, *TD* typical development.

**Table 5:** Statistical differences between the three groups.

	Odds ratio	*p* value
ASD above-the-mean vs. TD	0.914	0.861
ASD below-the-mean vs. TD	2.746	0.0102
ASD below-the-mean vs. above	2.506	0.027

*P* value is calculated by the two-sided Fisher’s exact test

In the post hoc exploratory analysis, we tested whether adding prenatal features and social behavior scores into models increases model performance. The Bayesian model AUC-ROC increased from 84.67 to 88.20% for the Training dataset, and increased from 89.18 to 91.48% for the Test dataset. (Supplementary Result 4).

## Discussion

Despite its high heritability and prenatal beginnings [1–16], ASD diagnosis remains behavior-based and the median age of the first diagnosis is about 52 months. Partially due to its genetic and clinical heterogeneity, no single genetic, behavioral or imaging diagnostic marker has been found that can accurately and reproducibly diagnose more than a small subset of affected children. Even among those capable of highly accurately diagnosing subsets of ASD infants and toddlers [82], few have proven clinically useful, cost-effect, and/or practical at the ages when early detection and diagnosis are most needed and could be most important for the child and family.

To approach this dilemma, we addressed ASD genetic and clinical heterogeneity with classifier heterogeneity. That is, since we expected heterogeneity in classifier gene expression features, we designed a classifier pipeline using 42,840 models generated from 3,570 gene expression feature routes and 12 classification methods to classify ASD at ages 1 to 4 years, and applied it to both a Training sample and a held-out Test sample. Then, rather than selecting and reporting a single “best” performing model, we report there are hundreds of good to excellent models and that they can be combined using Bayesian model averaging to bring together 742 “heterogeneous” predictive models involving 125 different feature routes and 2,721 gene expression features. The smMIPs analyses detected 25 TD and ASD subjects with severe mutations in SFARI Level 1 or 2 ASD risk genes: mutation-based classification resulted in chance ASD detection performance, whereas the Bayesian gene expression model correctly classified 22 (88%) of those 25 subjects. The presence of ASD risk gene mutations in typically developing toddlers suggests that the mutations detected here in these specific SFARI genes are neither necessary nor sufficient to cause ASD, are not alone explanatory of autism, and apparently are not clinically diagnostically useful.

Post-hoc Game-Howell tests demonstrated the ensemble gene expression classifier is unbiased towards age differences. The one-way ANOVA test indicated the classifier scores for the ASD group were similar across Hispanic and non-Hispanic subjects and different races. This suggests the classifier is accurately detecting a gene expression pathology common across toddler ages, races and ethnicities in ASD, subjects with and without risk gene mutations, and thus points to common core molecular pathobiology in ASD.

This approach enabled the generation of a composite Bayesian “ensemble” model that is diagnostically predictive and replicable across different toddler ages, races, and ethnicities; performs accurately across the ASD spectrum from more affected to less affected; and has potential for clinical translation. Moreover, this composite ensemble model incorporates both differentially expressed (DE) genes and non-DE genes. This may be relevant to the known complexity of ASD genetics, which may involve common and rare variants and any one or more of >200 different ASD risk gene mutations in different individuals. Non-genetic heterogeneity was also detected here insofar as those with ASD classifier scores below the overall ASD mean tended to have more prenatal risk events in their history than those ASD toddlers with above the mean scores. This opens the important potential to utilize these ASD ensemble classifier scores in future research to identify ASD subtypes that are more driven by genetic versus subtypes more driven by a combination of non-genetic and genetic factors.

Our ensemble features include genes involved in PI3K-AKT, RAS-ERK, and Wnt signaling pathways, immune/inflammation, response to cytokines, transcriptional regulation, and mitotic cell cycle, which are among the pathways and processes found across diverse studies on ASD blood gene expression [33–43, 45]. This overlap is notable despite the fact that (1) some previous studies did not actively account for race- and ethnicity-related, age-related or clinical-symptom heterogeneity as moderating factors; (2) 84% of 35 previous ASD blood gene expression studies had fewer than 100 ASD subjects and averaged only 28 ASD subjects/study; and (3) many studies focused on older ASD children and adults and only few on ASD toddlers [33–43].

PI3K-AKT, RAS-ERK and Wnt signaling pathways may be pivotal to ASD prenatal neural maldevelopment. Recently, in a large sample study, we discovered that ASD toddlers had significant upregulation of PI3K-AKT, RAS-ERK and Wnt signaling pathways in both leukocytes and iPSC-derived prenatal neural progenitors and neurons [45]. This leukocyte dysregulation in 1–4 year old ASD toddlers correlated with ASD social symptom severity [45]. Moreover, these pathways in leukocytes are downstream targets of regulatory risk ASD genes [3, 45]. Leukocyte gene expression also has an potential for understanding molecular correlates of brain size in ASD [33] and of atypical cortical patterning subtypes in ASD toddlers with poor language outcome outcomes [47]. Leukocyte expression also relates to hypoactivation response to affective speech in ASD toddlers with poor language outcome [46]. Finally, multivariate leukocyte expression signatures can predict trajectories of response to early intervention treatment [83], which underscores the relevance of leukocytes to ASD and clinically important phenomena that can be individualized to specific patients. Thus, extensive literature, meta-analyses, and the predictive diagnostic discoveries in the present study, all point to the importance of leukocyte cell biology as clinically informative in ASD and show that ASD-relevant dysregulated gene expression is not restricted to the brain but is also present in other tissues and organs.

Here we developed an innovative and accurate ASD gene expression classifier in ASD toddlers with heterogeneous gene features designed to address early-age ASD genetic and clinical heterogeneity. This predictive classifier in ASD male toddlers aged 1 to 4-year-olds opens the possibility of further refining ASD molecular classifiers optimized for race, ethnicity, and age and with potential for clinical utility. It far outperformed a risk gene-mutation classifier tested in the same toddlers primarily because a significant proportion of TD toddlers have ASD risk gene mutations as well. The ensemble gene expression ASD classifier reported here is enriched in gene expression features involved in ASD prenatal and postnatal pathobiology, and as such, it appears to succeed because of this. Thus, it is more than a signature capable of ASD diagnostic prediction; it is additionally a marker of the underlying pathobiological bases of the disorder in a majority of affected toddlers. It has implications for future research targeting early-age ASD detection and treatment-relevant mechanisms.

## Supplementary information

Supplementary material

Supplementary Tables 1 to 9

## Data Availability

We provide the code library in R and Python described in this work through Github: https://github.com/LewisLabUCSD/autism_classifier. We provide Jupyter notebooks in Python and R to generate our figures and analysis.
