# Supplementary material for: A predictive ensemble classifier for the gene expression diagnosis of ASD at ages 1 to 4 years
Source: Mol Psychiatry. Author manuscript; Available in PMC 2023 Mar 12. (PMC9908553; doi:10.1038/s41380-022-01826-x)
Supplement: Supplementary Figures and Analyses [file NIHMS1870186-supplement-Supplementary_Figures_and_Analyses.docx]

**A Predictive Ensemble Classifier for the Gene Expression Diagnosis of ASD at Ages 1 to 4 Years**

Bokan Bao^1,2,3,4,*^, Javad Zahiri^1*^, Vahid H. Gazestani^1,2*^, Linda Lopez^1^, Yaqiong Xiao^1,5^, Raphael Kim^6,7^, Teresa H. Wen^1^, Austin W.T. Chiang^1,2^, Srinivasa Nalabolu^1^, Karen Pierce^1^, Kimberly Robasky^7,8,9,10^, Tianyun Wang^11,12^, Kendra Hoekzema^13^, Evan E. Eichler^13,14^, Nathan E. Lewis^2,3,4,‡^, Eric Courchesne^1,‡^

### Affiliations

1. Autism Center of Excellence, Department of Neuroscience, University of California San Diego, La Jolla, CA, USA
   Javad Zahiri, Vahid H. Gazestani, Bokan Bao, Yaqiong Xiao, Teresa Wen, Srinivasa Nalabolu, Karen Pierce & Eric Courchesne
2. Department of Pediatrics, University of California San Diego, La Jolla, CA, USA
   Vahid H. Gazestani, Bokan Bao, Austin W.T. Chiang & Nathan E. Lewis
3. Bioinformatics and Systems Biology Program, University of California San Diego, La Jolla, CA, USA
   Bokan Bao & Nathan E. Lewis
4. Department of Bioengineering, University of California San Diego, La Jolla, CA, USA
   Bokan Bao & Nathan E. Lewis
5. Center for Language and Brain, Shenzhen Institute of Neuroscience, Shenzhen, China

Yaqiong Xiao

1. Department of Computer Science, University of North Carolina, Chapel Hill, NC, USA
   Raphael Kim
2. Renaissance Computing Institute, The University of North Carolina at Chapel Hill, Chapel Hill, NC, USA
   Raphael Kim & Kimberly Robasky
3. Department of Genetics, University of North Carolina at Chapel Hill, Chapel Hill, NC 27514, United States
   Kimberly Robasky
4. School of Information and Library Science, University of North Carolina at Chapel Hill, Chapel Hill, NC 27599, United States
   Kimberly Robasky
5. Carolina Health and Informatics Program, University of North Carolina at Chapel Hill, Chapel Hill, NC 27599, United States
   Kimberly Robasky
6. Department of Medical Genetics, Center for Medical Genetics, Peking University Health Science Center, Beijing 100191, China

Tianyun Wang

1. Neuroscience Research Institute, Peking University; Key Laboratory for Neuroscience, Ministry of Education of China & National Health Commission of China, Beijing 100191, China
   Tianyun Wang
2. Department of Genome Sciences, University of Washington School of Medicine, Seattle, WA 98195, USA
   Kendra Hoekzema & Evan E. Eichler
3. Howard Hughes Medical Institute, University of Washington, Seattle, WA 98195, USA

Evan E. Eichler

*These first authors contributed equally: Bokan Bao, Javad Zahiri, and Vahid H. Gazestani

‡ Corresponding co-equal Senior authors are Nathan E. Lewis and Eric Courchesne

Names: Nathan E. Lewis

Address: 9500 Gilman Drive MC 0760, La Jolla, CA 92093

E-mail: nlewisres@ucsd.edu

Name: Eric Courchesne

Address: 8110 La Jolla Shores Dr #201, La Jolla, CA 92037

E-mail: ecourchesne1949@gmail.com

**Content**

**eResult 1** Validation of Independent Replication dataset

**eResult 2** Result and parameter setting on the baseline Random Forest model

**Supplementary Figure 1**

**Supplementary Figure 2**

**Supplementary Figure 3**

**Supplementary Figure 4**

**Supplementary Figure 5**

**Supplementary Figure 6**

**Supplementary Figure 7**

**Supplementary Figure 8**

**Supplementary Method**

**1. The supplementary detail for the classifier pipeline’s feature selection and feature reduction**

On the second step (Supplementary Fig. 1), feature selection, included seven groups of methods. These seven groups are no (no action), grn[[1]](https://paperpile.com/c/KFPJBI/giiwr) (genetic regulatory network), z-score, selectV[[2]](https://paperpile.com/c/KFPJBI/ZqxLa), svm[[3]](https://paperpile.com/c/KFPJBI/gDG1i), GSEA[[4]](https://paperpile.com/c/KFPJBI/3037t), DE-analysis[[5]](https://paperpile.com/c/KFPJBI/HF9va). The Grn group has 14 methods, based on gene regulatory network analysis. The Z-score group has 4 methods for selecting features with high z-score variance. It picks the features with variance in the first quartile and then selects features within the 3rd quartile of the context likelihood of relatedness. The SelectV group has 54 methods and bases on the "Variable Selection for High-Dimensional Supervised Classification"[[2]](https://paperpile.com/c/KFPJBI/ZqxLa). In this group, features are first filtered by 'ExpHC','HC', 'Fair' methods (with/without comvar). The remaining features are further selected by the grn method with different parameters. The Svm group has 4 methods leveraging support vector machines with feature selection methods "scad", "L1", "ElasticNet" and "scad+L2". The GSEA group has 10 methods based on gene set enrichment analysis. The features are chosen with either FDR (False discovery rate)<0.1 or FDR<0.25. The features are ordered by FDR value within both feature groups and selected by choosing the lowest 10%, lowest 20%, lowest 50%, or below mean threshold. The remaining features are filtered by the cor and the grn3 method. The DE-analysis group has 15 methods and is picking features based on differential gene expression. The methods pick features with the combination of *P* value (*P* < 0.05 or *P* <0.01), fold change (logFc >0 or logFc<0 or both), and different numbers of features (1000, 500, or 100 features with the lowest *P*).

The third step was feature reduction (Supplementary Fig. 1). The seven methods include no (no feature reduction), WGCNA[[6]](https://paperpile.com/c/KFPJBI/wYunA), logisticFwd, SIS[[7]](https://paperpile.com/c/KFPJBI/rFFva), principal component regression (PCR)[[8]](https://paperpile.com/c/KFPJBI/sU2yB), partial least squares regression (PLSR)[[9]](https://paperpile.com/c/KFPJBI/Varyb), canonical powered partial least squares (CPPLS)[[9]](https://paperpile.com/c/KFPJBI/Varyb). The WGCNA, weighted correlation network analysis, calculates modules of co-expressed genes[[6]](https://paperpile.com/c/KFPJBI/wYunA), and we select hub genes from each module. The LogisticFwd method deploys stepwise logistic regression with the forward method to select features. The SIS[[7]](https://paperpile.com/c/KFPJBI/rFFva) method returns the features selected by the SIS R package, which implements the “Iterative Sure Independence Screening” for selecting variants. PCR regresses on a subset of principal components for dimensionality reduction , while PLSR finds a dimensionality-reducing linear regression model by projecting the variables to a new space, and CPPLS allows for discrete and continuous responses in the PLS model. After three steps, up to 1320 gene routes were created that can be used in the classification step.

**2. Visualization of the classifier AUC-ROC scores vs route mean AUC-ROC score**

The 12-classifier mean AUC-ROC scores of 1320 routes were calculated and set as x-axis. AUC-ROC scores of the 12-classifiers were visualized as y-axis (Supplementary Fig. 2c and d). Then, the 12-classifier mean AUC-ROC scores of 1320 routes were binned into 10 quantiles and the variance of the 12-classifiers AUC-ROC score for each route were calculated and presented as boxplot (Supplementary Fig. 2e and f).

**3. The gene set similarity score between routes**

To characterize the most common routes in classifiers with values of 0.80 or above, we compared the first five hundred genes that existed in those common routes (see Supplementary Table 5). In order to retrieve the genes from dimension reduction methods such pcr, cppls and plsr, we calculated the feature weights and the gene weights in each feature. Then, we ranked the genes with the weights they contribute to all features and selected the genes that cumulated to 66% of total weights.

- $-\log_{2} \mathrm{similarityScor}e_{i,j}=-\log_{2} \left[ \frac{\left( \left| overlapped gene \right|+1 \right)^{2}}{(\left| \mathrm{gen}\mathrm{eset}_{i} \right|+1)(\left| \mathrm{gen}\mathrm{eset}_{j} \right|+1)} \right]$

In the Supplementary Figure 4 and 5, the distance of the routes gene similarity is presented as
$-log_{2}\mathrm{similarityScor}e_{i,j}$.

**4. Post Hoc exploratory test combining GeoPref non-social test score and prenatal events as feature along with the ensemble features**

In a post hoc step, we tested whether adding the GeoPref eye tracking score and prenatal events along with the ensemble classification score would further improve overall classifier performance. GeoPref scores were available for 132 of the 175 Training subjects and 41 of the 65 of the subjects found in the independent Test dataset. The logistic regression with learning rate alpha=0.1 is used as the final model.

**Supplementary Result**

**1. High-performing models typically classify subjects similarly**

We next leveraged our set of 742 high-performing models to determine whether or not different classification models identified different subgroups of ASD toddlers. We clustered the 175 subjects in the Training dataset based on classification score similarity (Fig. 3a). The classification scores provide a probabilistic measure on the certainty of a model on the diagnostic status of each Training sample. It ranges from 0 to 1 with 0 being the highest certainty in TD status and 1 being the highest certainty in ASD status. The hierarchical clustering analysis found only one ASD group separated from TD subjects. This shows that the 742 models, overall, worked similarly in their assignment of classification scores to the ASD toddlers; that is, there was no evidence that different models selected different subgroups of ASD toddlers. This was also true in analysis of the holdout Test dataset.

**2. Genes driving classification enrich multiple processes consistent with the ASD literature**

We characterized the feature routes of 742 models showing AUC-ROC above 0.8 in the Discovery dataset and above 0.80 in the independent Replication dataset. The 742 models mostly originated from 125 feature routes.

To identify the biological processes that contribute to these 125 feature routes, we scored all genes in these routes based on the number of times they occurred in different 125 routes (Supplementary Table 5). We found that the top 500 scoring genes selected by the feature routes had a larger impact on the model performance than the 12 classifiers. Different routes that largely overlapped in selected feature genes also showed highly correlated performance across different classifier methods (see Methods, Supplementary Fig. 4 and 6). We next calculated the enrichment of biological processes among 500 most common feature genes using g:Profiler. In this work, the 500 most common genes were enriched (Supplementary Table 7) in the KEGG pathways of inflammation/immune related disease, including Acute myeloid leukemia (KEGG: hsa05221), Measles infection (KEGG: hsa01524) and other significant pathways such as oxidative phosphorylation (KEGG: hsa04070), Ras signaling pathway (KEGG: has04014) and Wnt signaling pathway (KEGG: hsa04310). Similarly, enriched Gene Ontology terms for biological processes were associated with the down-regulation in blood inflammation/immune cell response, transcriptional gene regulation and response to cytokines, which were consistent with previous studies[[10–12]](https://paperpile.com/c/KFPJBI/gxhjt+Chse5+j6FnN) (see Methods).

**3. Result and parameter setting on the baseline Random Forest model**

For this estimate of AUC-ROC baseline, the Test dataset was randomly split into a separate “baseline-validation” and “baseline-test” set. The same training dataset was used for training. Using the Training set, we performed feature selection for the top ***q*** variables, as scored by the chi-squared statistic. From a grid, ***q*** was chosen based on which grid value yielded the highest mean AUC after 100 random forest evaluations on the “baseline-validation” set. Additionally, we averaged the feature importance rankings over the 100 iterations to provide a more comprehensive view of important genes. With the selected ***q*** value, we trained a random forest classifier and evaluated on the held-out “baseline-test” set. Number of top variables are chosen from results based on the validation data, over a grid of (10, 100, 500, 1000). On this grid, 500 genes are chosen as optimal. Importances are generated from 100 rounds of evaluations of Random Forest, averaged. Final test results using these top 500 genes have the accuracy: 73.33%, sensitivity: 85.71%, specificity: 62.50%, AUC-ROC: 72.32%.

**4. Post Hoc exploratory test: Combining GeoPref (non-social) test score and prenatal effects as features along with ensemble features**

Since social attention abnormalities are core ASD symptoms and visual social attention deficits well described[[13–15]](https://paperpile.com/c/KFPJBI/Un0kt+MGs0L+WQqTm), in a post hoc test we tested whether adding a social feature to the molecular ensemble would increase classifier performance. For this, we used toddlers' gaze fixation score on non-social (i.e., geometric) images as measured in an eye-tracking test known as the GeoPref Test[[13–15]](https://paperpile.com/c/KFPJBI/Un0kt+MGs0L+WQqTm). GeoPref scores were available for 132 of the 175 Training subjects and 41 of the 65 of the subjects found in the held out Test dataset. Based on their prior GeoPref test results, subjects who had percent fixation on non-social images >69% were labeled as being the ASD GeoPref-subtype[[13–15]](https://paperpile.com/c/KFPJBI/Un0kt+MGs0L+WQqTm) (Supplementary Fig. 6). With prenatal effects and eye-fixation features added, the Bayesian model AUC-ROC increased from 84.67% to 88.20% for the Training dataset and increased from 89.18% to 91.48% for the Test dataset.

**Supplementary Figure 1. The detailed workflow of the feature engineering pipeline.**
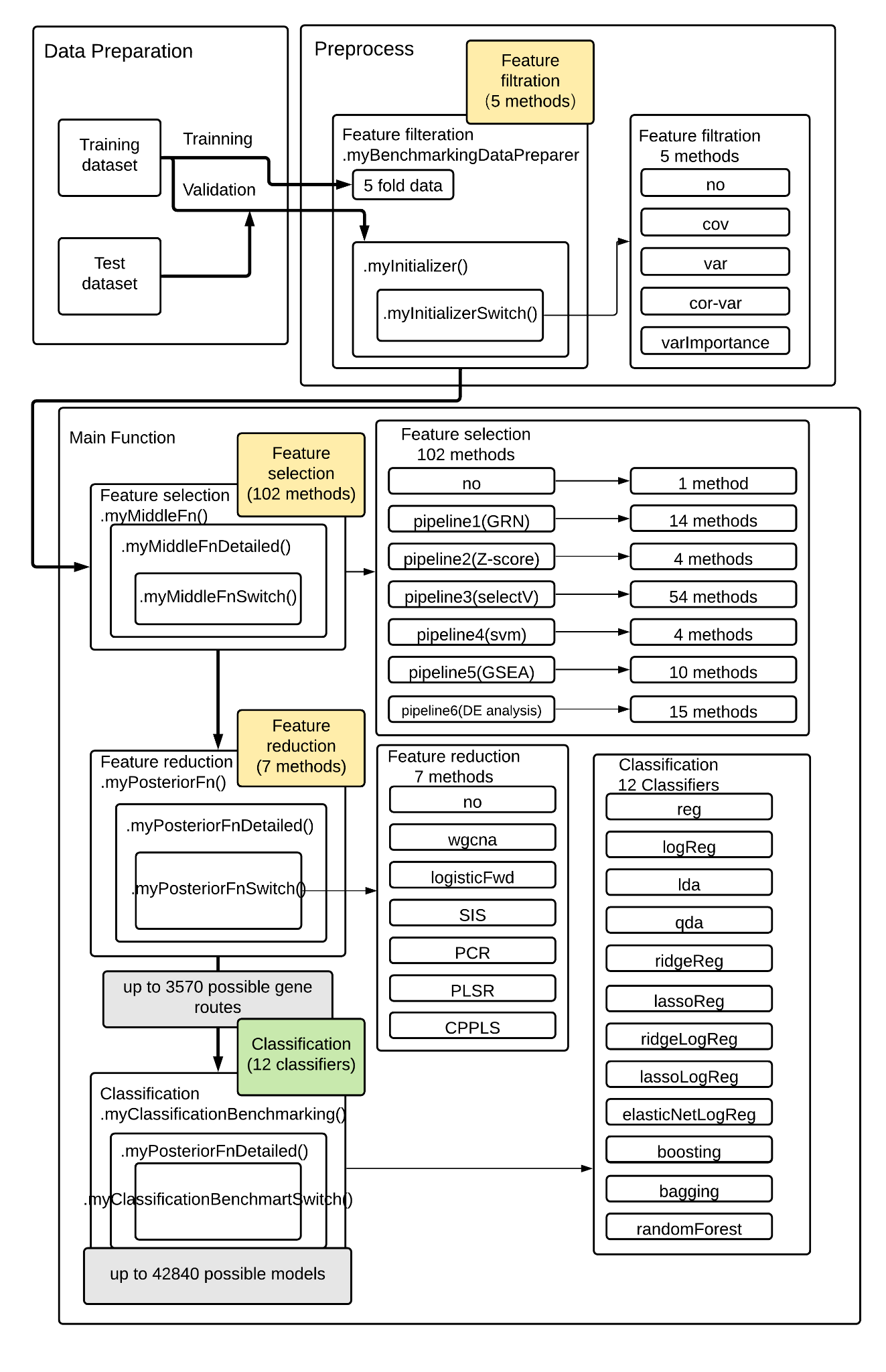


**Supplementary Figure 2. The distribution of AUC-ROC scores of 12 classifiers against the AUC-ROC mean score of 1320 routes.** The distribution of AUC-ROC generated by 1320 routes within each of 12 classifiers in **a** the discovery dataset and **b** the independent dataset. The distribution of AUC-ROC generated by 1320 routes vs the mean of AUC-ROC of each route in **c** the discovery dataset (with +- 0.0514 95% confidence interval) and **d** the distribution in the independent dataset (with +- 0.0514 95% confidence interval) . The variance of AUC-ROC generated by 12 classifiers of 1320 routes, the x-axis is the mean of AUC-ROC of each route in **e** the main dataset and **f** in the test dataset.


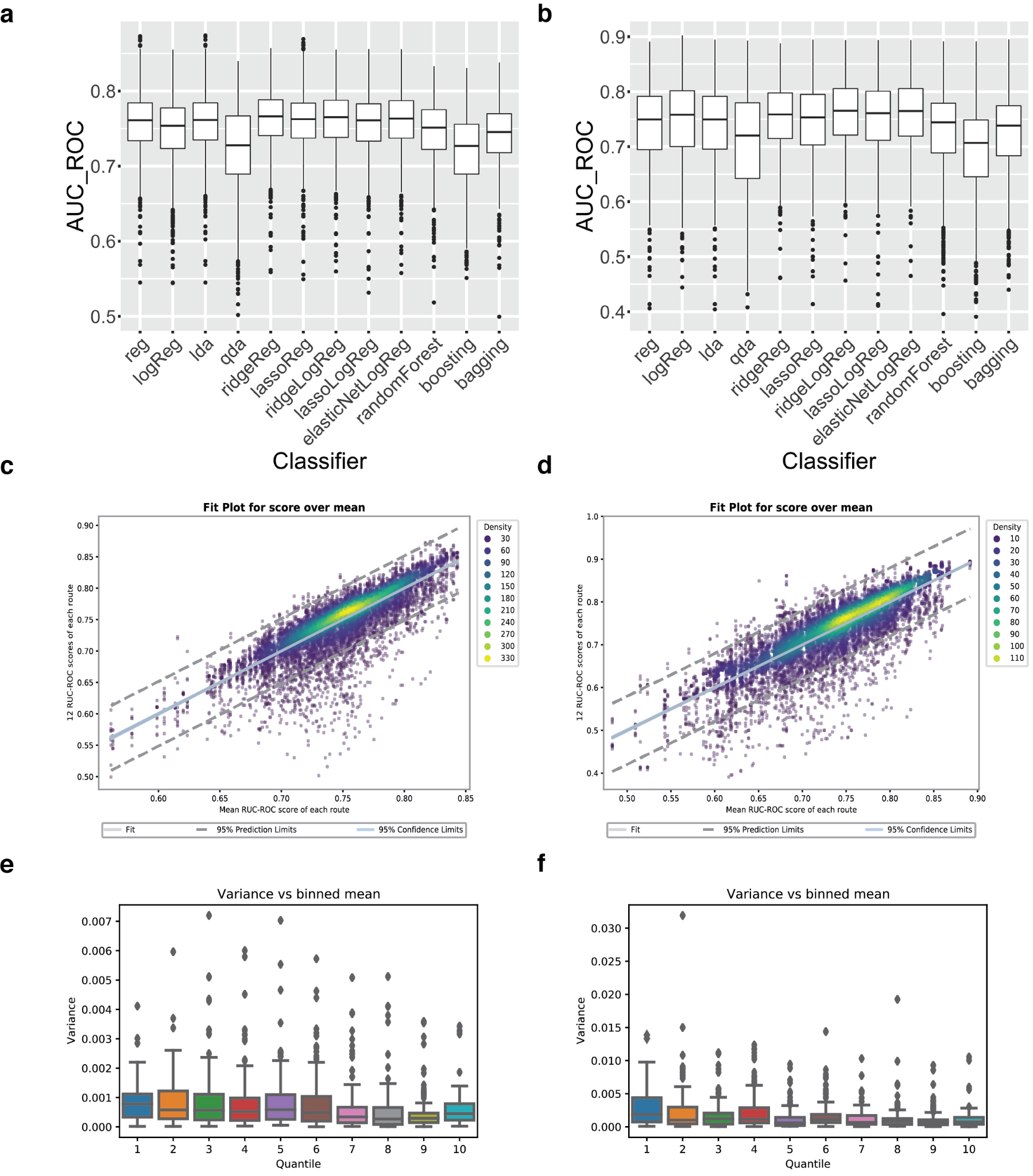


**Supplementary Figure 3. The AUC-ROC score of 1320 models trained on Discovery dataset. a** The score distribution of 1822 models that have AUC-ROC score above 0.8 in the discovery dataset in 5 iterations. **b** The score-difference distribution of 1822 models in 5 iterations.


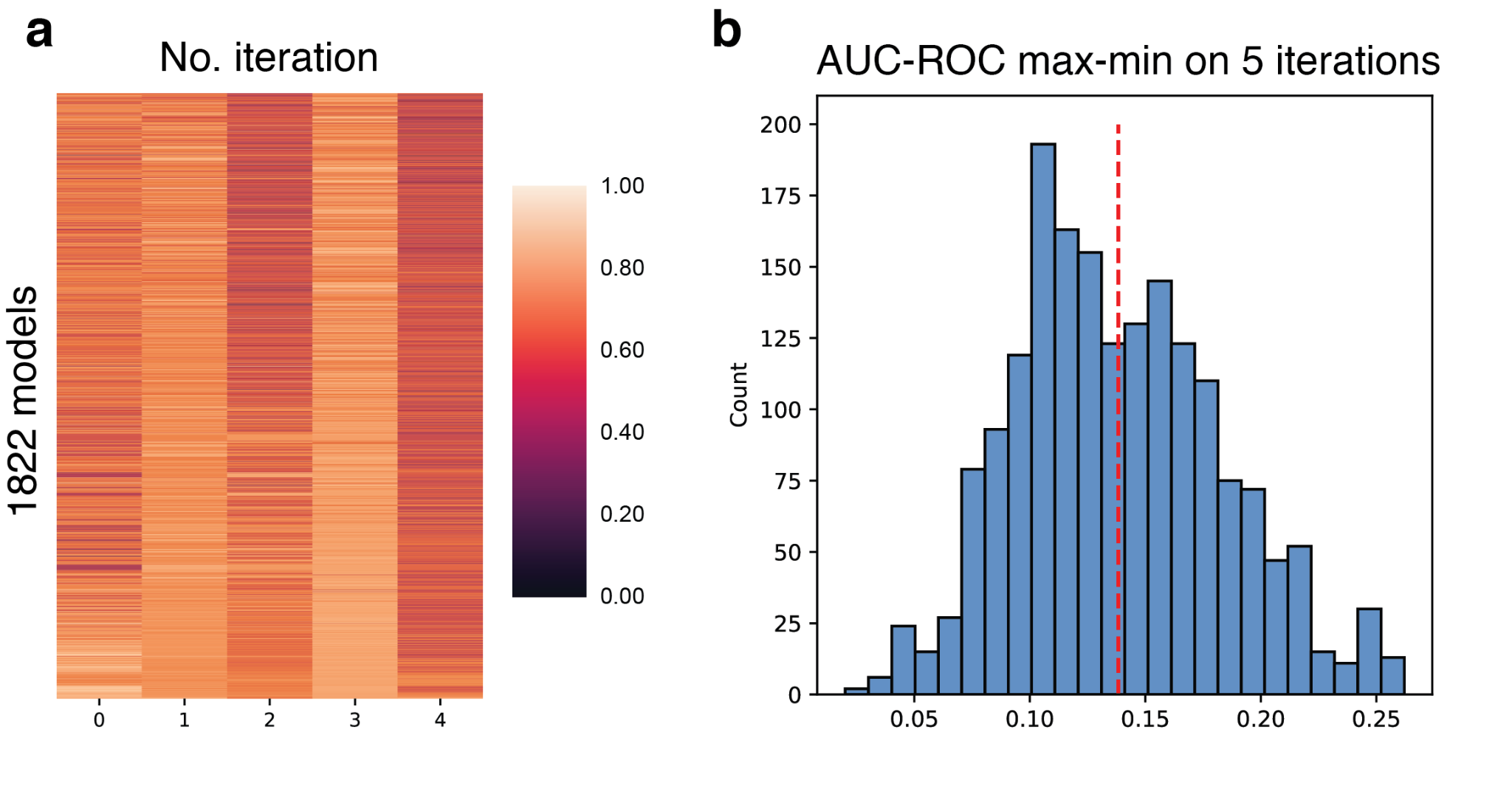


**Supplementary Figure 4**

**The geneset similarity distance among 125 routes.** The distance score (Supplementary Method 3) is measured by the top 500 genes that are used by 125 routes and then clustered by the ‘average’ method. The data of the table is in supplementary table 9.


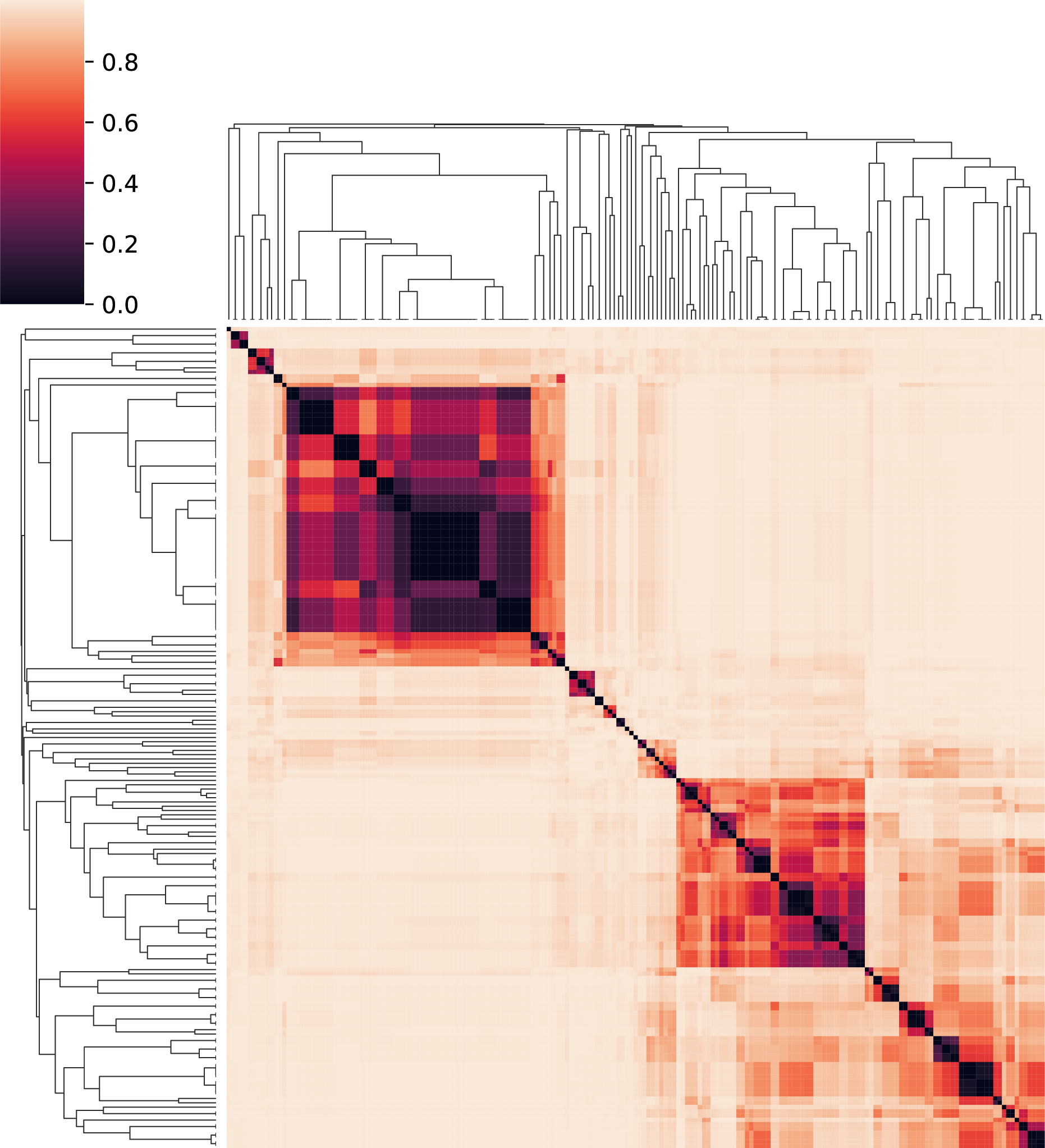


**Supplementary Figure 5. The relation between the similarity of gene routes and the 12 classifiers behavior.**

The correlation of 12-classifier score vs the distance between routes and models. The distance is measured by the -log2 of the geneset similarity (Supplementary Method 3) on the first 500 genes. The

slope=-0.03806, intercept=0.4444, rvalue=-0.2529, *p* = 1.354x10^-26^, stderr=0.001075


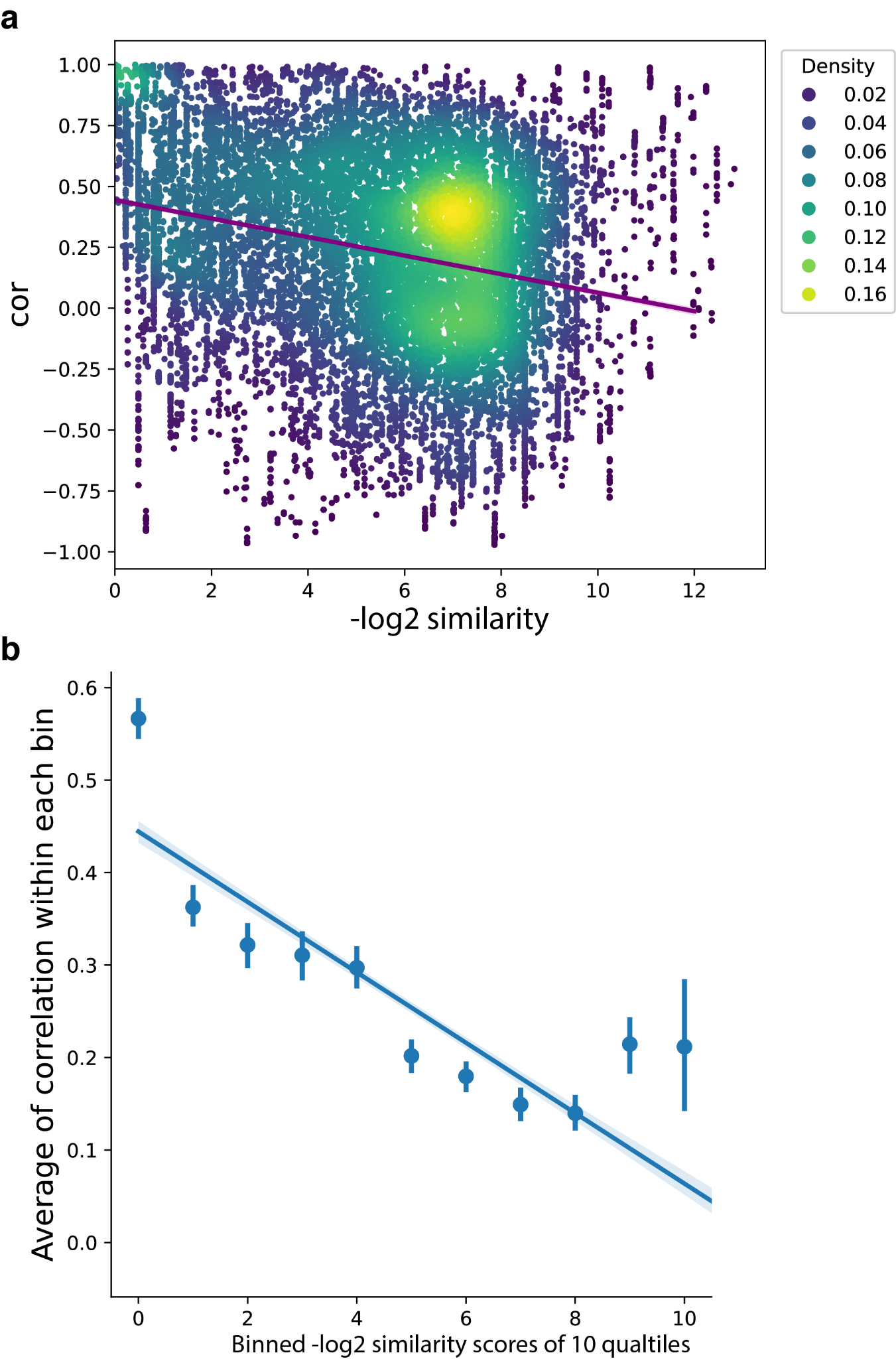


**Supplementary Figure 6. Illustration of the updated model that combines the composite model with geo-fixation model.** The updated model was tested on 132 of 175 Discovery dataset subjects and 41of 65 Replication dataset subjects who had available Geo-Fixation data (e.g., moderate or good data quality, total looking time > 50%). By directly classifying the subjects who had percent fixation on non-social images >69% as ASD (GeoPref-subtype). **a** The composite score vs geo-fixation percentage score in the training dataset. **b** The composite score vs geo-fixation percentage score for the test dataset. **c** The updated score vs geo-fixation percentage score for the training dataset. **d** The updated score vs geo-fixation percentage score for the test dataset.

**
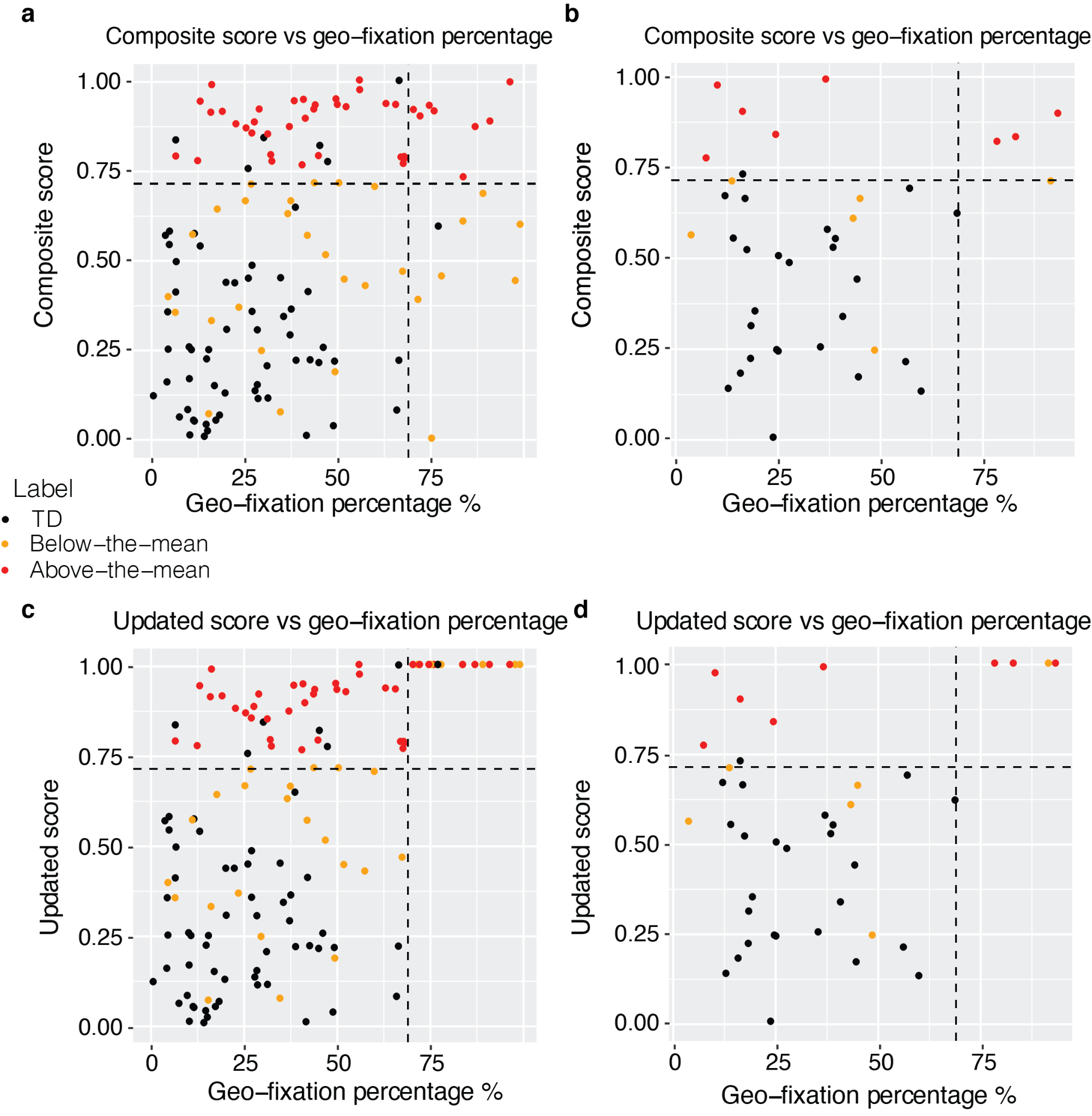
**

**Supplementary Figure 7. Comparison of ASD with and without ASD risk gene mutations and TD with and without ASD risk gene mutations.** Comparing ensemble classifier scores, ADOS (Severity) scores (higher scores are more severe ASD symptoms), Vineland adaptive behavior scores (Adaptive), and Mullen T-scores for Receptive and Expressive language as well as the Mullen overall Developmental Quotient scores (Devel. Quotient). There were no significant differences in any scores between toddlers with and without SFARI Level 1 or 2 ASD risk gene mutations for ASD toddlers and for TD toddlers (at level of FDR<0.05).

**Supplementary Figure 8.** Diagnostic and psychometric scores were not significantly different between ASD toddlers above (above-the-mean) and below the mean composite classifier score (below-the-mean).

**a** two sided t.test *p* = 4.011x10^-14^. **b** training dataset r^2^ = 0.306, coefficient=0.026

**c** two sided t.test *p* = 3.873x 10^-5^. **d** test dataset r^2^ = 0.210, coefficient=0.019


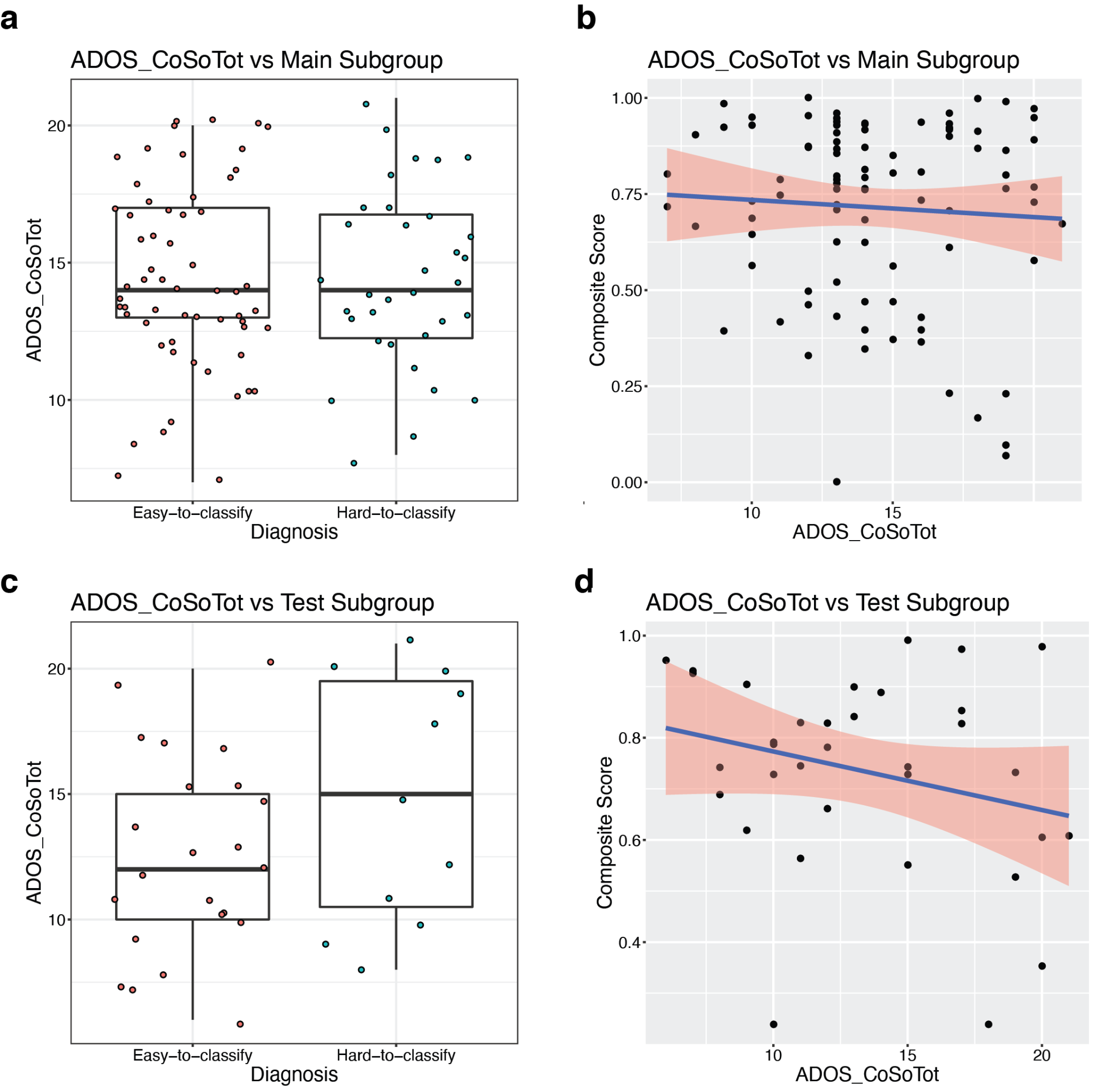


**Reference**

1. [Meyer PE, Lafitte F, Bontempi G. minet: A R/Bioconductor package for inferring large transcriptional networks using mutual information. BMC Bioinformatics. 2008;9:461.](http://paperpile.com/b/KFPJBI/giiwr)

2. [Antonio Pedro Duarte Silva <psilva@porto.ucp.pt>. SelectV: Variable selection for high-dimensional supervised... In HiDimDA: High dimensional Discriminant Analysis. 2015.](http://paperpile.com/b/KFPJBI/ZqxLa) <https://rdrr.io/cran/HiDimDA/man/SelectV.html.> [Accessed 21 May 2021.](http://paperpile.com/b/KFPJBI/ZqxLa)

3. [penalizedSVM: Feature Selection SVM using Penalty Functions.](http://paperpile.com/b/KFPJBI/gDG1i) <https://cran.r-project.org/web/packages/penalizedSVM/index.html.> [Accessed 29 June 2021.](http://paperpile.com/b/KFPJBI/gDG1i)

4. [Subramanian A, Tamayo P, Mootha VK, Mukherjee S, Ebert BL, Gillette MA, et al. Gene set enrichment analysis: a knowledge-based approach for interpreting genome-wide expression profiles. Proc Natl Acad Sci U S A. 2005;102:15545–15550.](http://paperpile.com/b/KFPJBI/3037t)

5. [Ritchie ME, Phipson B, Wu D, Hu Y, Law CW, Shi W, et al. limma powers differential expression analyses for RNA-sequencing and microarray studies. Nucleic Acids Research. 2015;43:e47–e47.](http://paperpile.com/b/KFPJBI/HF9va)

6. [Langfelder P, Horvath S. WGCNA: an R package for weighted correlation network analysis. BMC Bioinformatics. 2008;9:559.](http://paperpile.com/b/KFPJBI/wYunA)

7. [Saldana DF, Feng Y. SIS: An R Package for Sure Independence Screening in Ultrahigh-Dimensional Statistical Models. Journal of Statistical Software, Articles. 2018;83:1–25.](http://paperpile.com/b/KFPJBI/rFFva)

8. [Mevik B-H, Wehrens R. Introduction to the pls Package. Help Section of The ‘Pls’ Package of R Studio Software; R Foundation for Statistical Computing: Vienna, Austria. 2015:1–23.](http://paperpile.com/b/KFPJBI/sU2yB)

9. [Wehrens R, Mevik B-H. The pls package: principal component and partial least squares regression in R. 2007. 2007.](http://paperpile.com/b/KFPJBI/Varyb)

10. [Pramparo T, Pierce K, Lombardo MV, Carter Barnes C, Marinero S, Ahrens-Barbeau C, et al. Prediction of autism by translation and immune/inflammation coexpressed genes in toddlers from pediatric community practices. JAMA Psychiatry. 2015;72:386–394.](http://paperpile.com/b/KFPJBI/gxhjt)

11. [Kong SW, Collins CD, Shimizu-Motohashi Y, Holm IA, Campbell MG, Lee I-H, et al. Characteristics and predictive value of blood transcriptome signature in males with autism spectrum disorders. PLoS One. 2012;7:e49475.](http://paperpile.com/b/KFPJBI/Chse5)

12. [He Y, Zhou Y, Ma W, Wang J. An integrated transcriptomic analysis of autism spectrum disorder. Sci Rep. 2019;9:11818.](http://paperpile.com/b/KFPJBI/j6FnN)

13. [Pierce K, Conant D, Hazin R, Stoner R, Desmond J. Preference for geometric patterns early in life as a risk factor for autism. Arch Gen Psychiatry. 2011;68:101–109.](http://paperpile.com/b/KFPJBI/Un0kt)

14. [Moore A, Wozniak M, Yousef A, Barnes CC, Cha D, Courchesne E, et al. The geometric preference subtype in ASD: identifying a consistent, early-emerging phenomenon through eye tracking. Mol Autism. 2018;9:19.](http://paperpile.com/b/KFPJBI/MGs0L)

15. [Pierce K, Marinero S, Hazin R, McKenna B, Barnes CC, Malige A. Eye Tracking Reveals Abnormal Visual Preference for Geometric Images as an Early Biomarker of an Autism Spectrum Disorder Subtype Associated With Increased Symptom Severity. Biol Psychiatry. 2016;79:657–666.](http://paperpile.com/b/KFPJBI/WQqTm)
